# Assessment of hESC‐IMRC‐Exo for Cardiac and Cerebral Injuries Post‐Cardiac Arrest Resuscitation: Safety, Pharmacokinetics, and Efficacy

**DOI:** 10.1111/jcmm.71264

**Published:** 2026-06-26

**Authors:** Huijuan Yang, Xiaodan Zhang, Wenbin Zhang, Xue Wang, Jie Wang, Ziwei Chen, Jinyu Zhu, Yufeng Hu, Lu He, Licai Liang, Jiefeng Xu, Mao Zhang

**Affiliations:** ^1^ Department of Emergency Medicine, Second Affiliated Hospital Zhejiang University School of Medicine Hangzhou China; ^2^ Zhejiang Key Laboratory of Trauma, Burn, and Medical Rescue Hangzhou China; ^3^ Zhejiang Province Clinical Research Center for Emergency and Critical Care Medicine Hangzhou China; ^4^ Institute of Biotechnology, Xianghu Laboratory Hangzhou Zhejiang China; ^5^ Oujiang Laboratory Wenzhou China

**Keywords:** cardiac arrest, cardiac injury, cardiopulmonary resuscitation, cerebral injury, immunity and matrix regulatory cell‐derived exosomes

## Abstract

Cardiac arrest (CA) is a leading cause of death globally. Exosomes derived from mesenchymal stem cells exhibit favourable production, storage, and safety characteristics, making them a promising alternative for post‐CA resuscitation. We assessed the safety, biodistribution, and protective effects of human embryonic stem cell‐derived immunity‐and‐matrix regulatory cells (hESC‐IMRC‐Exo) for post‐CA resuscitation cardiac/cerebral injuries, and preliminarily explored its underlying molecular mechanism. The biotoxicity of IMRC‐Exo was evaluated in AC16 and HT22 cells and in mice; its biodistribution was traced using fluorescently labelled IMRC‐Exo. Protective effects were examined in H/R‐treated cells and rat and swine CA models. We found that IMRC‐Exo did not induce apoptosis or oxidative stress in AC16 or HT22 cells. IMRC‐Exo was also safe in mice, with normal body weight, blood indices, and histopathology. Pharmacokinetic analysis revealed rapid multi‐organ distribution, peaking at 24 h with a 7‐day half‐life. For efficacy study, IMRC‐Exo reduced LDH release, ROS levels, and apoptosis in H/R‐treated cells. In rat, IMRC‐Exo dose‐dependently (0.5×, 1×, 2×) reduced serum biomarkers, improved neurological function, and attenuated inflammation. In swine, single‐dose IMRC‐Exo (1×) reduced injury biomarkers, improved neurological function, and attenuated inflammation. Furthermore, in a separate rat group, IMRC‐Exo improved 7‐day survival and neurological function. Mechanistically, the protective effects of IMRC‐Exo were mediated by its secreted miR‐21‐5p, which attenuated H/R‐induced apoptosis and inflammation by targeting PDCD4. This study demonstrated IMRC‐Exo, a safe and effective therapeutic, distributed rapidly via the bloodstream to target organs and protected against post‐resuscitation cardiac/cerebral injuries, potentially through the miR‐21‐5p/PDCD4 axis.

**Abbreviations**
CA: cardiac arrest
CPR: cardiopulmonary resuscitation
cTnI: cardiac troponin I
EBs: embryoid bodies
ECG: electrocardiogram
GEF: global ejection fraction
H/R: hypoxia/reoxygenation
hESC‐IMRC‐Exo: exosomes secreted from human embryonic stem cell‐derived immunity‐and‐matrix regulatory cells
IgE: immunoglobulin E
IL‐1β: interleukin‐1β
IL‐6: interleukin‐6
IMRCs: immunity and matrix regulatory cells
MSC‐Exo: MSC‐derived exosomes
MSCs: mesenchymal stem cells
NSE: neuron‐specific enolase
PCAS: post‐cardiac arrest syndrome
PLTs: platelets
RBCs: red blood cells
ROS: reactive oxygen species
S100β: S100β protein
SV: stroke volume
TNF‐α: tumour necrosis factor alpha
VF: ventricular fibrillation
WBCs: white blood cells

## 1 Introduction

Cardiac arrest (CA), a frequent clinical emergency, has emerged as a leading cause of death and disability globally. Recent survey data have revealed that the annual incidence of out‐of‐hospital CA in the United States is 88.8 cases per 100,000 individuals, with a hospital discharge survival rate of only 9.3% [1, 2]. Notably, China has an annual incidence of out‐of‐hospital CA of approximately 95.7 cases per 100,000 individuals, with a hospital discharge survival rate of as low as 1.15% [3]. Consequently, seeking effective treatment modalities for improving the survival rates of patients with CA has caused widespread concern.

As a significant cause of death following successful resuscitation from CA, post‐CA syndrome (PCAS) originating from global ischemia–reperfusion (I/R) injury caused by the resuscitation clinically manifests as cardiac and cerebral organ damage, along with systemic multi‐organ dysfunction [4, 5]. Despite international guidelines recommending treatment modalities, including therapeutic hypothermia, these therapies have been proven insufficient to significantly improve clinical prognosis for cardiac and cerebral injuries during PCAS [6]. Moreover, several promising therapeutic approaches in laboratory settings have not yet been successfully translated into clinical applications [7, 8, 9, 10]. In this context, developing new‐generation treatment strategies that are both effective and clinically feasible for the efficient treatment of cardiac and cerebral injuries following cardiopulmonary resuscitation (CPR) is urgently needed.

Growing clinical evidence has indicated that mesenchymal stem cells (MSCs) possess omnifarious biological effects, including anti‐inflammatory, antioxidant, and anti‐apoptotic properties, which can reduce I/R injury to the whole body and enhance the clinical outcomes of patients with CA [11, 12, 13, 14]. Of note, compared with the existing guideline‐recommended strategy of therapeutic hypothermia for neuroprotection, preliminary research has revealed that adipose‐derived MSCs exhibit superior roles in enhanced anti‐inflammatory effects, intensive antioxidant properties, and improved neuronal survival [15]. Moreover, human embryonic stem cell (hESC)‐derived immunity and matrix regulatory cells (IMRC) possess unparalleled preponderance in terms of cell quality, immune regulation, and tissue repair capabilities compared with those derived from conventional adult tissues, such as bone marrow, umbilical cord, and adipose tissues. Consequently, this cell‐based therapy potentially becomes a powerful means of improving neurological abnormalities following CPR. Based on this aforementioned research evidence, we further observed that IMRC exerted a positive protective effect on reducing CA‐induced cardiac and cerebral injuries in swine models. Moreover, MSC‐derived exosomes (MSC‐Exo), as pivotal mediators of MSC therapy for various acute diseases (e.g., I/R injury), possess the semblable biological effects of MSCs and are easier to access, store, and safer, which are anticipated to be an excellent alternative to MSC therapy [16, 17]. However, studies on the effects of exosomes secreted from human embryonic stem cell‐derived immunity‐and‐matrix regulatory cells (hESC‐IMRC‐Exo) in the treatment of cardiac and cerebral injuries following CA remain lacking.

This study aimed to evaluate the feasibility of IMRC‐Exo for the management of cardiac and cerebral injuries following CPR. Subsequently, the biosafety, pharmacokinetics, and efficacy of these nanovesicles were evaluated. Consequently, this study established a scientific foundation for the clinical application of exosomes, in anticipation of improving prognosis for patients with CA.

## 2 Materials and Methods

### 2.1 IMRC‐Exo Production and Isolation

**FIGURE 1:**
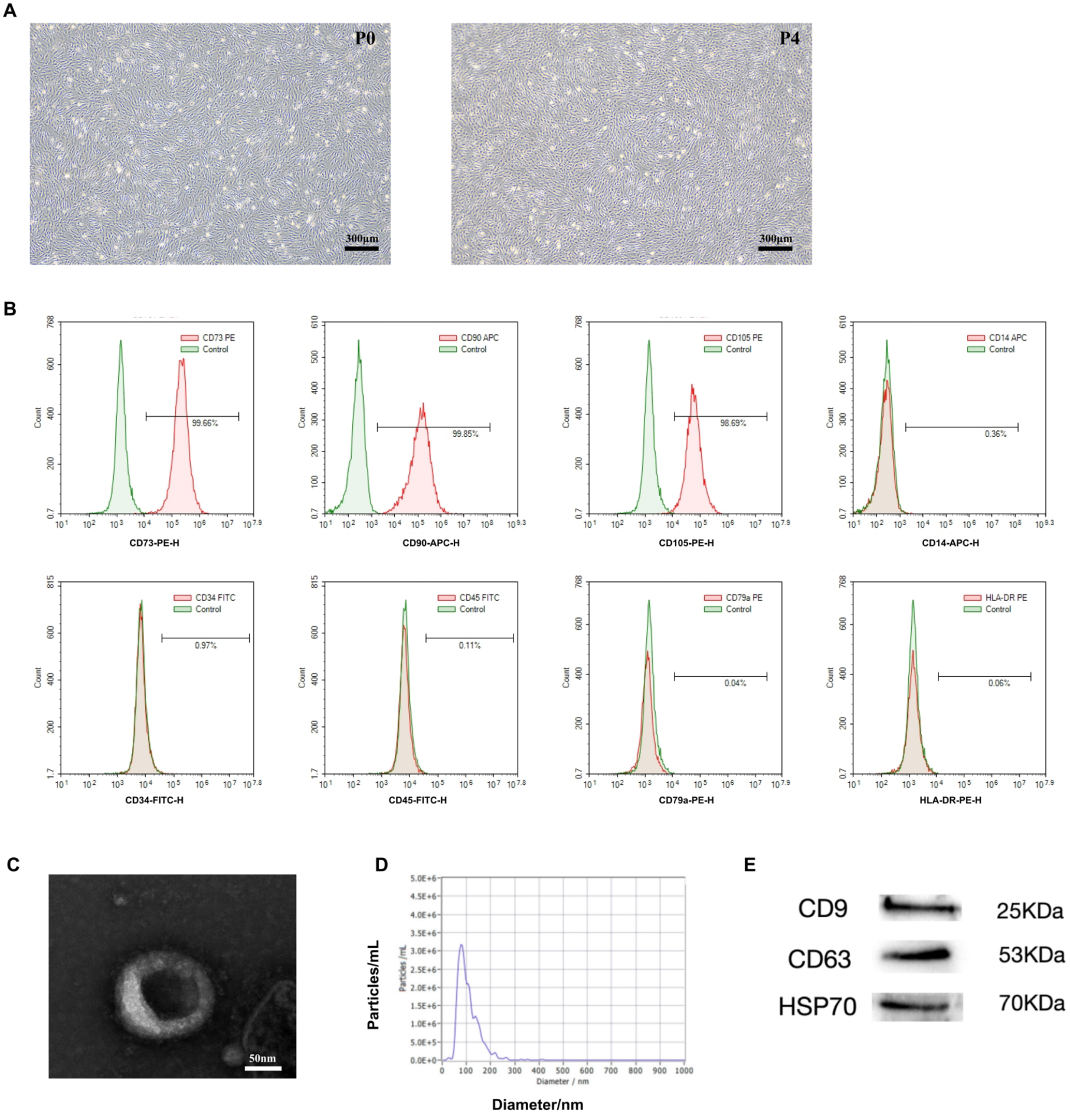
IMRC‐Exo characterisation. (A) Representative micrographs for the morphological characterisation of IMRC at P0 and P4. Scale bar: 300 μm. (B) Flow cytometry analysis for specific surface antigen markers of IMRC at P4. (C) Representative image of IMRC‐Exo captured using TEM. Scale bar: 100 nm. (D) IMRC‐Exos are analysed using NTA. E HSP70, CD63, and CD9 expression in IMRC‐Exo is determined using Western blotting.

IMRCs were derived from hESCs (the Stem Cell Bank of the Institute of Biochemistry and Cell Biology, Shanghai Institutes for Biological Sciences, Chinese Academy of Sciences) via a two‐step process [12, 18]. Briefly, H9‐hESCs were dissociated with TrypLE Express and cultured in NutriStem hPSC XF medium (Bioindustry, 05‐100‐1A) on ultra‐low adhesion plates for 5 days to form embryoid bodies (EBs). Next, EBs were cultured in an IMRC‐induced differentiation medium (Luyuan Biotech, LYIMRC‐4001) for 11 days and subsequently subcultured with TrypLE Express. Following subculturing, cells were continuously cultured in an IMRC‐induced differentiation medium until reaching 90% confluence, at which point they were defined as passage 0 (P0), characterised by a uniform spindle‐shaped cell population. Afterward, IMRCs were expanded to passage 4 (P4) in a chemically defined proliferation medium (Luyuan Biotech, LYIMRC‐4002) and subsequently used in animal experiments, with their morphology at P0 and P4 shown in Figure 1A.

IMRC phenotypes were analysed using flow cytometry (Agilent, NovoCyte 2060R) [12]. Cells were dissociated, suspended in fluorescence‐activated cell sorting buffer (1× PBS/0.5% BSA), and incubated with fluorescently labelled antibodies for 30 min at 4°C. Data were collected using Agilent NovoCyte and analysed using NovoExpress (Figure 1B).

hESCs‐IMRCs were cultured in a chemically defined proliferation medium. When the cell confluence reached 80%, the supernatant was collected and centrifuged at 4000 *g* for 20 min to remove cell debris. Tangential flow filtration (Sartorius VF05H4, 100 kDa) was employed for processing the cell supernatant at a 50:1 concentration ratio, achieving ultrafiltration and concentration of the sample. Subsequently, a filter membrane with a 0.22‐μm pore size was used to further remove any smaller particulates and impurities. The concentrated and filtered supernatant was purified using size exclusion chromatography (IZON, qEV10), and the obtained IMRC‐Exos were collected. To confirm the size distributions of these nanovesicles, nanoparticle tracking analysis (NTA) (Particle Metrix, ZetaView) was performed, followed by imaging analysis using Hitachi transmission electron microscopy (TEM), and Western blot analysis was conducted to detect surface marker proteins of IMRC‐Exos (Figure 1C–E).

### 2.2 Cell Experiments

HT22 cell line (BNCC358041, mouse hippocampal neuronal cell line) and AC16 cell line (BNCC339980, human cardiomyocytes) were both purchased from the Shanghai Meixuan Biotechnology Co. Ltd. HT22 and AC16 cells were cultured in high‐glucose Dulbecco's Modified Eagle Medium (DMEM) containing 10% fetal bovine serum.

For safety evaluation, normal HT22 and AC16 cells were cocultured with PBS and 100 μg/mL of IMRC‐Exo under standard conditions for 24 h. Subsequently, the apoptosis ratio and reactive oxygen species (ROS) levels were measured.

For efficacy evaluation, cells were divided into the following three groups: control, hypoxia/reoxygenation (H/R), and H/R + IMRC‐Exo groups. Cells remained untreated in the control group. For hypoxia induction, HT22 cells were incubated in glucose‐free DMEM in a hypoxic chamber with 1% O_2_, 5% CO_2_, and 94% N_2_ for 10 h, and AC16 cells underwent a similar treatment for 8 h. Subsequently, cells were incubated at 37°C in high‐glucose DMEM medium and reoxygenated for 24 h. During the reoxygenation period, the H/R + IMRC‐Exo group was cocultured with IMRC‐Exo at a 100‐μg/mL concentration, whereas the H/R group was cocultured with an equivalent volume of PBS. Subsequently, cell viability, lactate dehydrogenase (LDH) levels, ROS levels, and apoptosis ratios were monitored.

To determine whether IMRC‐Exo protects against H/R injury by delivering miR‐21‐5p to target PDCD4, rescue experiments were performed in HT22 and AC16 cells. MiR‐21‐5p mimics and the negative control were synthesised by Quanyang Biotechnology (Shanghai, China). The PDCD4 overexpression plasmid and the empty vector were constructed by Quanyang Biotechnology (Shanghai, China). All were stored and diluted according to the manufacturer's instructions. Cells were divided into five groups: NC group: normal culture without H/R treatment or transfection; H/R group: H/R model established, transfected with the empty vector plasmid and miRNA NC; H/R + miR‐21‐5p mimics group: transfected with the empty vector plasmid and miR‐21‐5p mimics at 48 h prior to H/R treatment; H/R + oePDCD4 group: transfected with the PDCD4 overexpression plasmid and miRNA NC at 48 h prior to H/R treatment; H/R + miR‐21‐5p mimics + oePDCD4 group: co‐transfected with miR‐21‐5p mimics and the PDCD4 overexpression plasmid at 48 h prior to H/R treatment. The miRNAs, plasmids, and Lipofectamine 3000 (Thermo Fisher Scientific, Waltham, MA, USA) were separately diluted in antibiotic‐free and serum‐free Opti‐MEM medium, mixed at the ratio recommended by the manufacturer to form transfection complexes, and added to the cell culture plates. The plates were gently rocked to ensure even distribution of the complexes over the cell surface. At 6 h post‐transfection, the medium was replaced with fresh complete medium. At 48 h post‐transfection, the cells were subjected to H/R treatment.

### 2.3 Animal Experiments

The Institutional Ethics Committee of the Second Affiliated Hospital of Zhejiang University School of Medicine granted the ethical approval for the animal experiments outlined in this study. Healthy C57BL/6 mice, aged 8–10 weeks and weighing approximately 25 g, were purchased from Shanghai SLAC Laboratory Animal Co. Ltd. (Shanghai, China); healthy male Wistar rats, aged 10–14 weeks and weighing 350–400 g, were obtained from Zhejiang Vital River Laboratory Animal Co. Ltd. (Zhejiang, China); and healthy male swine, aged 4–6 months and weighing 39 ± 2 kg, were acquired from Shanghai Jiagan Biotechnology Inc. (Shanghai, China).

All animals were maintained under the standard conditions, including standard atmospheric pressure, room temperature (20°C–25°C), humidity (60%–80%), 12/12‐h light/dark cycle, closed cages, spontaneous access to water, regular feeding, and routine cleaning with disinfection.

### 2.4 Mice Model

For the in vivo analysis of exosomal toxicity, healthy C57BL/6 mice were randomly assigned to five groups and subsequently received intravenous injection of PBS or IMRC‐Exo (2.5 × 10^10^, 5 × 10^10^, 1.25 × 10^11^, and 2.5 × 10^11^ particles/kg) via the tail vein (*n* = 6 mice per group). Mice were anaesthetised by intraperitoneal administration of 1% sodium pentobarbital (50 mg/kg). At the designated time points (0, 1, 3, 7, and 14 days), body weights were recorded, and 100–200‐μL blood samples were collected via eye puncture. Routine blood tests and blood biochemical analyses were subsequently conducted, assessing red blood cells (RBCs), white blood cells (WBCs), platelets (PLTs), lymphocytes, eosinophils, basophils, interleukin‐6 (IL‐6), and total immunoglobulin E (IgE). Furthermore, at 14 days post‐injection, all mice were euthanised by intraperitoneal injection of 1% pentobarbital (150 mg/kg), followed by collection of vital organs (heart, brain, liver, spleen, lungs, kidneys, and intestines) for haematoxylin and eosin (H&E) staining.

For biodistribution evaluation, IMRC‐Exos were labelled with a fluorescent dye for exosome labeling (Umibio Science and Technology Group) following the manufacturers' instructions and subsequently intravenously administered into the mice (*n* = 5) via the tail vein. Before tail vein injection, mice received intraperitoneal anaesthesia with 1% sodium pentobarbital (50 mg/kg). The hair on the mouse chest, abdomen, or back was shaved and depilated as necessary. Whole‐body fluorescence intensity was recorded at 1 h, 3 h, 6 h, 12 h, 7 days, and 14 days post‐injection utilizing an AniView multimodal imaging system. For each session, to ensure immobilisation, mice were anaesthetised with 2% isoflurane (1 L/min O_2_). Metabolic curves were plotted on the basis of the collected data to evaluate IMRC‐Exo in vivo distribution. On account of the observed IMRC‐Exo in vivo distribution patterns, we selected 1, 3, 6, and 24 h as key time points for further analysis, with five mice per time point receiving the fluorescently labelled IMRC‐Exo. Whole‐body fluorescence imaging was performed at these time points. Subsequently, euthanasia was induced by pentobarbital overdose, followed by harvest of major organs (heart, brain, kidneys, liver, lungs, and intestines) for ex vivo fluorescence imaging.

### 2.5 Rat Model

As previously described [10], animals were fasted overnight with access to water and subsequently anaesthetised using sodium pentobarbital at a 45‐mg/kg dosage (with supplementary doses of 10 mg/kg hourly for maintenance). A 14‐gauge cannula was inserted through the mouth into the trachea to facilitate mechanical ventilation. Subcutaneous needle electrodes were attached to the limbs for continuous recording of lead II electrocardiogram (ECG), whereas a polyethylene catheter (PE50, RWD, China) was inserted into the femoral vessels for arterial pressure monitoring. Blood pressure and ECG data were collected using the BL‐420 N data acquisition and analysis system (Chengdu Taimeng Software Co. LTD, Chengdu, China), with a heating lamp employed to maintain a rectal temperature of 36.8°C ± 0.4°C.

Rats were randomly assigned into five groups (*n* = 5 per group): Sham, CPR, CPR + 0.5× IMRC‐Exo, CPR + 1× IMRC‐Exo, and CPR + 2× IMRC‐Exo. The standard dose of IMRC‐Exo was 2.5 × 10^10^ particles/kg. Accordingly, rats in the 0.5×, 1×, and 2× IMRC‐Exo groups received 1.25 × 10^10^, 2.5 × 10^10^, and 5.0 × 10^10^ particles/kg via tail vein injection immediately after successful resuscitation, respectively. The sham and CPR groups simultaneously received the equivalent volume of normal saline. In the sham group, rats underwent surgical procedures without subsequent CA or resuscitation. The CPR and CPR + IMRC‐Exo groups underwent 6 min of ventricular fibrillation (VF)‐induced CA, followed by a 6‐min manual CPR. After a 2‐min CPR, rats received a bolus of 20‐μg/kg adrenaline, followed by repeat doses every 2 min. Defibrillation (Mindray Corporation, Shenzhen, China) attempts were performed at 6‐min intervals. Successful resuscitation was defined as the return of a supraventricular rhythm and an aortic systolic pressure of > 50 mmHg for at least 5 min. If this was not achieved, chest compressions and mechanical ventilation were promptly resumed, followed by defibrillation following an additional 2 min. This cycle was repeated up to three times until resuscitation was successful or deemed to be a failure. Following successful resuscitation, animals were monitored for 4 h before being returned to their cages for an additional 20 h of observation. At the end of the experiments, rats were euthanised via intraperitoneal injection of three times the dose of pentobarbital sodium (150 mg/kg) for a continuous 2–3 min.

For the long‐term effect study, rats were randomly divided into three groups: Sham, CPR, and CPR + IMRC‐Exo groups. All rats were maintained for 7 consecutive days after successful resuscitation.

### 2.6 Swine Model

Animals were fasted overnight with free access to water. As previously outlined [19], anaesthesia was induced using midazolam (0.4–0.5 mg/kg) and maintained with a continuous infusion of propofol (4 mg/kg/h). Animals were intubated and ventilated using a ventilator (Monnal T75, Air Liquide Medical Systems, Antony Cedex, France), and end‐tidal CO_2_ levels were monitored using a monitor/defibrillator (M Series, ZOLL Medical Corporation, Chelmsford, MA, USA). Two fluid‐filled 7‐Fr thermodilution‐tipped catheters were vein into the thoracic aorta and the right atrium to measure aortic and right atrial pressures and collect arterial and venous blood samples. A 4‐Fr thermistor‐tipped arterial catheter and a 7‐Fr central venous catheter were inserted into the left femoral artery and the right internal jugular vein, respectively, and both of them were connected to the PiCCO system (PiCCO plus, Pulsion Medical Systems, Munich, Germany) to monitor stroke volume (SV) and global ejection fraction (GEF). To induce ventricular tachycardia, a 5‐Fr pacing catheter was introduced into the right ventricle. Heart rate and mean arterial pressure were continuously recorded using the patient monitoring system (iM60, Edan, Shenzhen, China). Baseline arterial pH and lactate concentrations were determined using a blood gas analyser (i15, Edan, Shenzhen, China). Rectal temperature was maintained at 38.0°C ± 0.5°C using the Blanketrol III Hyper‐Hypothermia System (Cincinnati Sub‐Zero, Cincinnati, OH, USA).

Animals were randomly assigned into the following three groups: sham (*n* = 6), CPR (*n* = 6), and CPR + IMRC‐Exo (*n* = 6) groups. In the CPR + IMRC‐Exo group, swine underwent intravenous injection via the internal jugular vein with IMRC‐Exo at a dose of 2.5 × 10^10^ particles/kg within 30 min following successful resuscitation. Meanwhile, the sham and CPR groups received the equal volume of normal saline. The sham group only underwent surgery preparation without CA or resuscitation. The other two groups experienced 12 min of VF‐induced CA, followed by a 6‐min manual CPR. Following a 2‐min CPR, swine were treated with 20 μg/kg of epinephrine, with repeat doses administered at 3‐min intervals. Defibrillation attempts were made at 6‐min intervals. Successful resuscitation was deemed as an organised rhythm with a mean arterial pressure of > 50 mmHg persisting for at least 5 min. If this was not achieved, CPR was resumed for an additional 2 min before another electrical shock. This cycle was repeated up to five times until resuscitation was successful or affirmed to have failed. Following successful resuscitation, animals were monitored for 4 h before returning to their cages for an additional 20‐h observation. At the end of the experiments, swine were euthanised by intravenous injection of excessive pentobarbital sodium (100 mg/kg).

### 2.7 Cell Viability Assay

HT22 and AC16 cell activities were assessed using Cell Counting Kit‐8 (CCK‐8) (Biyuntian Biotechnology, Shanghai, China). Cells were seeded in 96‐well plates at a density of 5 × 10^3^ cells/well. Following H/R injury, the CCK‐8 reagent was added to each well at 10% of the total volume, and the plates were incubated at 37°C for 2 h in the dark. Subsequently, the optical density (OD) at a 450‐nm wavelength was detected using a microplate reader (Multiskan MK3, Thermo Fisher Scientific, Waltham, MA, USA).

### 2.8 ROS Assay

Intracellular ROS levels within HT22 and AC16 cells were measured using an ROS assay kit (Solarbio, Beijing, China). Cells were seeded in six‐well plates at a density of 2 × 10^5^ cells/well. Following H/R injury, cells were washed three times with PBS and subsequently incubated with 2‐μM 2′,7′‐dichlorodihydrofluorescein diacetate at 37°C for 20 min. The medium was discarded, and cells were washed three times with a high‐glucose DMEM medium before being observed under a fluorescence microscope. The fluorescence intensity was detected using a fluorescent microplate reader.

### 2.9 LDH Release Assay

The levels of LDH released from HT22 and AC16 cells were measured using an LDH assay kit (Abcam, Cambridge, MA, USA). Cells were seeded in six‐well plates at a density of 2 × 10^5^ cells/well. Following H/R injury, cells were lysed with 1% Triton X‐100. Following the manufacturer's instructions, substrate buffer, Coenzyme I, and 2,4‐dinitrophenylhydrazine were added. Subsequently, sodium hydroxide was added to the mixture for further incubation at room temperature for 5 min, and the OD values at the 450‐nm wavelength were detected using a microplate reader (Multiskan MK3, Thermo Fisher Scientific, Waltham, MA, USA).

### 2.10 Cell Apoptosis Detection

For apoptosis detection, cells were cultured in six‐well plates at a seeding density of 2 × 10^5^ cells/well. Following H/R treatment completion, cells and their corresponding supernatants were harvested. Subsequently, 10 μL of propidium iodide and 5 μL of Annexin V‐FITC stain were added, followed by incubation at room temperature for 5 min. Cell apoptosis was then detected using flow cytometry (BD Biosciences, San Jose, CA, USA) and analysed using FlowJo software (FlowJo LLC, Ashland, OR, USA).

### 2.11 Cell ELISA

Cell culture supernatants were collected and centrifuged at 1000× *g* for 15 min. The supernatants were added to ELISA plates pre‐coated with TNF‐α and IL‐1β antibodies (Anxuda Biotechnology Co. Ltd., Shanghai, China). Absorbance at 450 nm was measured using a microplate reader (Multiskan SkyHigh, MA, USA), and the concentrations of TNF‐α and IL‐1β were calculated by standard curve interpolation.

### 2.12 Western Blot

Cells were lysed in RIPA buffer, and protein concentrations were determined by BCA assay. Equal amounts of protein were separated by SDS‐polyacrylamide gel electrophoresis (SDS‐PAGE) and transferred to PVDF membranes. After blocking with 5% BSA at room temperature for 30 min, membranes were incubated with primary antibodies overnight at 4°C, followed by incubation with HRP‐conjugated secondary antibody at room temperature for 1 h. Protein bands were visualised using ECL reagent, and signals were captured using a chemiluminescence imaging system. Band intensities were analysed with ImageJ software.

The primary antibodies used in this study were as follows: rabbit anti‐PDCD4 (Proteintech, Wuhan, China, Catalogue #12587‐1‐AP, 1:1000), rabbit anti‐Cleaved Caspase 3 (Proteintech, Wuhan, China, Catalogue #25128‐1‐AP, 1:1000), rabbit anti‐p‐NF‐κB p65 (Proteintech, Wuhan, China, Catalogue #82335‐1‐RR, 1:1000), and rabbit anti‐GAPDH (Proteintech, Wuhan, China, Catalogue #10494‐1‐AP, 1:5000).

### 2.13 Luciferase Reporter Assay

To verify the binding relationship between miR‐21‐5p and mouse Pdcd4 or human PDCD4, wild‐type or mutant Pdcd4 3′‐UTR and PDCD4 3′‐UTR sequences were separately cloned into the psiCHECK2.0 vector (TongYong Bio, Anhui, China). HEK‐293 T cells were then transiently co‐transfected with the psiCHECK2.0 plasmids together with miR‐21‐5p mimics or NC. At 48 h post‐transfection, Firefly and Renilla luciferase activities were detected using the Dual‐Luciferase Reporter Assay System (Promega, Madison, WI, USA) according to the manufacturer's instructions. Luminescence values were measured using a multifunctional fluorescence microplate reader (Tecan, Männedorf, Switzerland). Finally, the relative Firefly luciferase activity of mPDCD4 or hPDCD4 was normalised to Renilla luciferase activity. The wild‐type and mutant 3′‐UTR sequences and the predicted miR‐21‐5p binding sites are presented in Figures S1 and S2.

### 2.14 Haematological Analysis

Blood samples were collected for haematological analysis at 0, 1, 3, 7, and 14 days following IMRC‐Exo injection into the mice. Blood was preserved in tubes with sodium ethylenediaminetetraacetic acid. WBC, RBC, PLT, neutrophil, and lymphocyte counts were investigated using an automatic haematological analyser (Mindray Corporation, Shenzhen, China). IL‐6 and IgE levels were measured using enzyme‐linked immunosorbent assay (ELISA) kits (Meixuan Biotechnology Inc., Shanghai, China) according to the manufacturers' instructions.

### 2.15 H&E Staining

Pathological analysis was performed at 14 days following IMRC‐Exo injection. Mice were euthanised, and major organs, including the heart, brain, liver, spleen, lung, kidney, and intestine, were harvested. These organs were fixed overnight in 4% paraformaldehyde, embedded in paraffin, and sectioned at a 3‐μm thickness. Next, these tissue sections were stained with H&E to observe the degree of lesions and inflammatory cell infiltration under an optical microscope at 100× magnification.

### 2.16 Myocardial Function and Cardiac and Cerebral Injury Assessment

Cardiac functions, indicated by the indexes of SV and GEF, were assessed using the PiCCO system at baseline, 1, 2, 4, 6, and 24 h following resuscitation. Moreover, cardiac and cerebral injury biomarkers, including cardiac troponin I (cTnI), neuron‐specific enolase (NSE), and S100β protein (S100β), were detected at the same time points. These biomarkers were determined from venous blood samples collected at the aforementioned intervals, with serum obtained for detection analysis using ELISA kits, following the manufacturers' instructions.

### 2.17 Cardiac and Cerebral Apoptosis and Inflammatory Cytokine Detection

Apoptosis in cardiac and cerebral tissues was performed at 24 h post‐resuscitation using TdT‐mediated dUTP nick end labeling (TUNEL) assay kit (Boster Biological Technology Co., Wuhan, China) following standard instructions. Briefly, the left ventricular apex, cerebral cortex, and hippocampus were immediately harvested following euthanasia and subsequently fixed in 4% paraformaldehyde for 24 h. Thereafter, these tissues were embedded in paraffin and finally sectioned into 5‐μm‐thick slices. These specimens were stained using the TUNEL assay kit. Next, six views were randomly selected to calculate the numbers of TUNEL‐positive cells and total cells at 200× magnification under an optical microscope (Biological microscope C×31, Olympus, Tokyo, Japan). The rate of apoptotic cells was calculated as the percentage of TUNEL‐positive cells relative to total cells.

The levels of tumour necrosis factor alpha (TNF‐α) and IL‐1β within cardiac and cerebral tissue samples at 24 h post‐resuscitation were measured using ELISA analyses. Subsequent to cardiac and cerebral tissue collection, these samples underwent homogenisation in normal saline over ice, followed by centrifugation at 4000 rpm at 4°C for 15 min. The resultant supernatants were collected to measure TNF‐α and IL‐1β levels.

### 2.18 Neurologic Deficit Score Evaluation and Survival Analysis

The neurologic function of swine in each group was evaluated using the neurological deficit score (NDS) and cerebral performance category (CPC) at 24 h post‐resuscitation. The NDS ranges from 0 (no neurologic deficit) to 400 (death or brain death), whereas the CPC score ranges from 1 to 5 [20, 21]. For rats in each group, the neurological function score (NFS) was assessed using two different scoring methods, with NFS‐1 and NFS‐2 scores ranging from 0 to 80 and 0 to 500 points, respectively [22, 23]. Survival curves were plotted using the Kaplan–Meier method over the 7‐day observation period and rats surviving to day 7 were subjected to neurological functional assessments. Significantly, two investigators who were blinded to the group allocations performed these assessments.

### 2.19 Statistical Analysis

Continuous variable distributions were assessed using the Kolmogorov–Smirnov test. Normally distributed data were expressed as means ± standard deviations (SDs) and evaluated using one‐way analysis of variance (ANOVA). Non‐normally distributed data were expressed as medians (interquartile ranges) and analysed utilizing the Kruskal‐Wallis test. Repeated measures ANOVA was performed for within‐group time‐based comparisons. Post hoc comparisons were conducted using the Bonferroni test when significant differences were noted. Survival curves were plotted using the Kaplan–Meier method, and between‐group survival rates were compared using the log‐rank test. All data were analysed using Statistical Package for the Social Sciences (version 20, IBM, Armonk, NY, USA).

## 3 Results

### 3.1 IMRC‐Exo Biosafety Assessment

**FIGURE 2:**
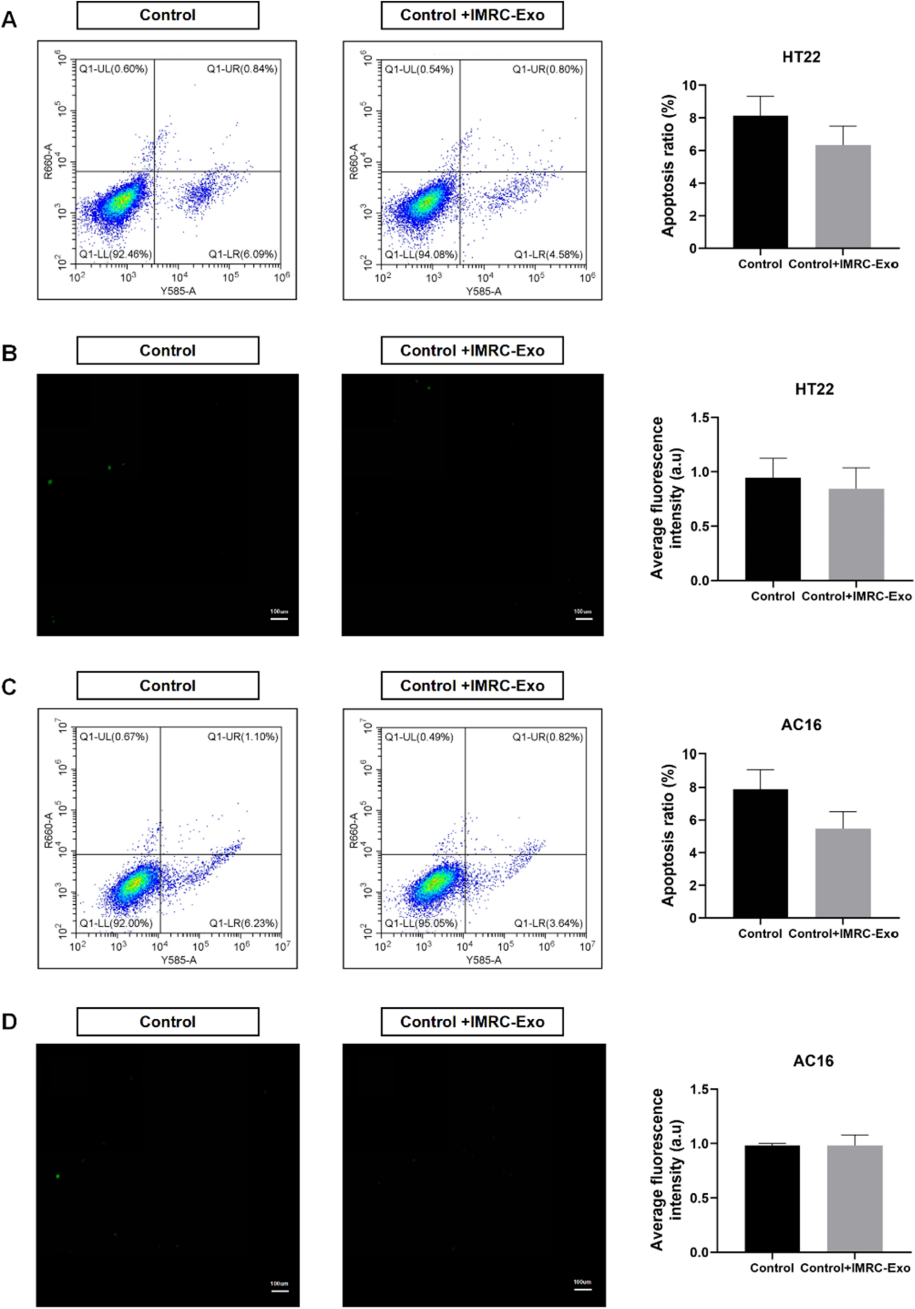
Effects of IMRC‐Exo on HT22 and AC16 cells under normal conditions. (A) Flow cytometric analysis and quantification of cell apoptosis in HT22 cells (*n* = 3). (B) Representative fluorescence images and quantification of ROS production in HT22 cells (*n* = 3). (C) Flow cytometric analysis and quantification of cell apoptosis in AC16 cells (*n* = 3). (D) Representative fluorescence images and quantification of ROS production in AC16 cells (*n* = 3). Data are presented as means ± SDs. *p < 0.05 versus the control group.

The biosafety of IMRC‐Exo was a prerequisite for implementing its clinical applications. In this context, we evaluated the biotoxicity of IMRC‐Exo by co‐incubation with two types of normal cells, AC16 and HT22. Following a 24‐h co‐incubation, IMRC‐Exo treatment did not induce apoptosis or oxidative damage in either cell type (Figure 2).

**FIGURE 3:**
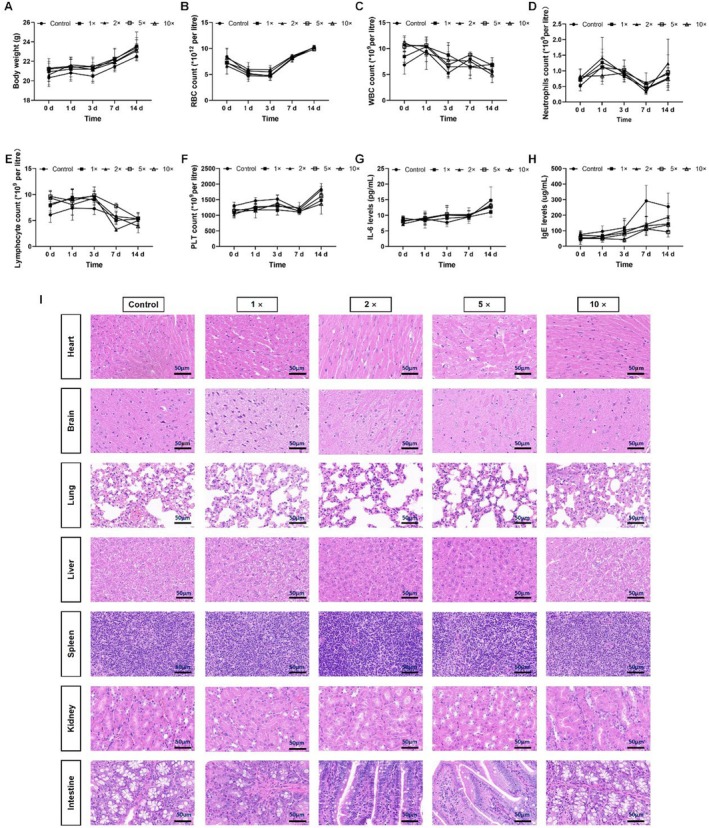
Effects of IMRC‐Exo on haematological parameters and organ pathology. (A–H) RBCs, WBCs, PLTs, lymphocytes, eosinophils, basophils, IL‐6, and total IgE evaluated at 0, 1, 3, 7, and 14 days following IMRC‐Exo injection (*n* = 6). (I) Representative images (100×) of H&E staining of vital organs at 14 days following IMRC‐Exo injection (*n* = 6). Scale bar: 50 μm. 1× (2.5 × 10^10^ particles/kg), 2× (5.0 × 10^10^ particles/kg), 5× (1.25 × 10^11^ particles/kg), and 10× (2.5 × 10^11^ particles/kg). Data are presented as means ± SDs.

Besides validating its safety in in vitro experiments, we proceeded to assess the in vivo biotoxicity of IMRC‐Exo by intravenous injection into healthy mice with various concentrations (2.5 × 10^10^, 5 × 10^10^, 1.25 × 10^11^, and 2.5 × 10^11^ particles/kg). The body weight change, routine blood indices, and histopathological alterations in the vital organs further underwent detection analysis. No significant difference was noted between the body weights of IMRC‐Exo–treated mice and the control group (Figure 3). Furthermore, no obvious fluctuations were observed in the proportions of inflammatory cells, including RBC, WBC, PLT, lymphocytes, eosinophils, and basophils, as well as the levels of pro‐inflammatory cytokines (IL‐6 and IgE), in the peripheral blood of IMRC‐Exo–treated mice, with all values remaining within the normal range. Additionally, IMRC‐Exo administration did not cause severe inflammatory infiltration and substantial tissue damage in these organs, aligning with the findings in the control group (Figure 3).

### 3.2 IMRC‐Exo Biodistribution

**FIGURE 4:**
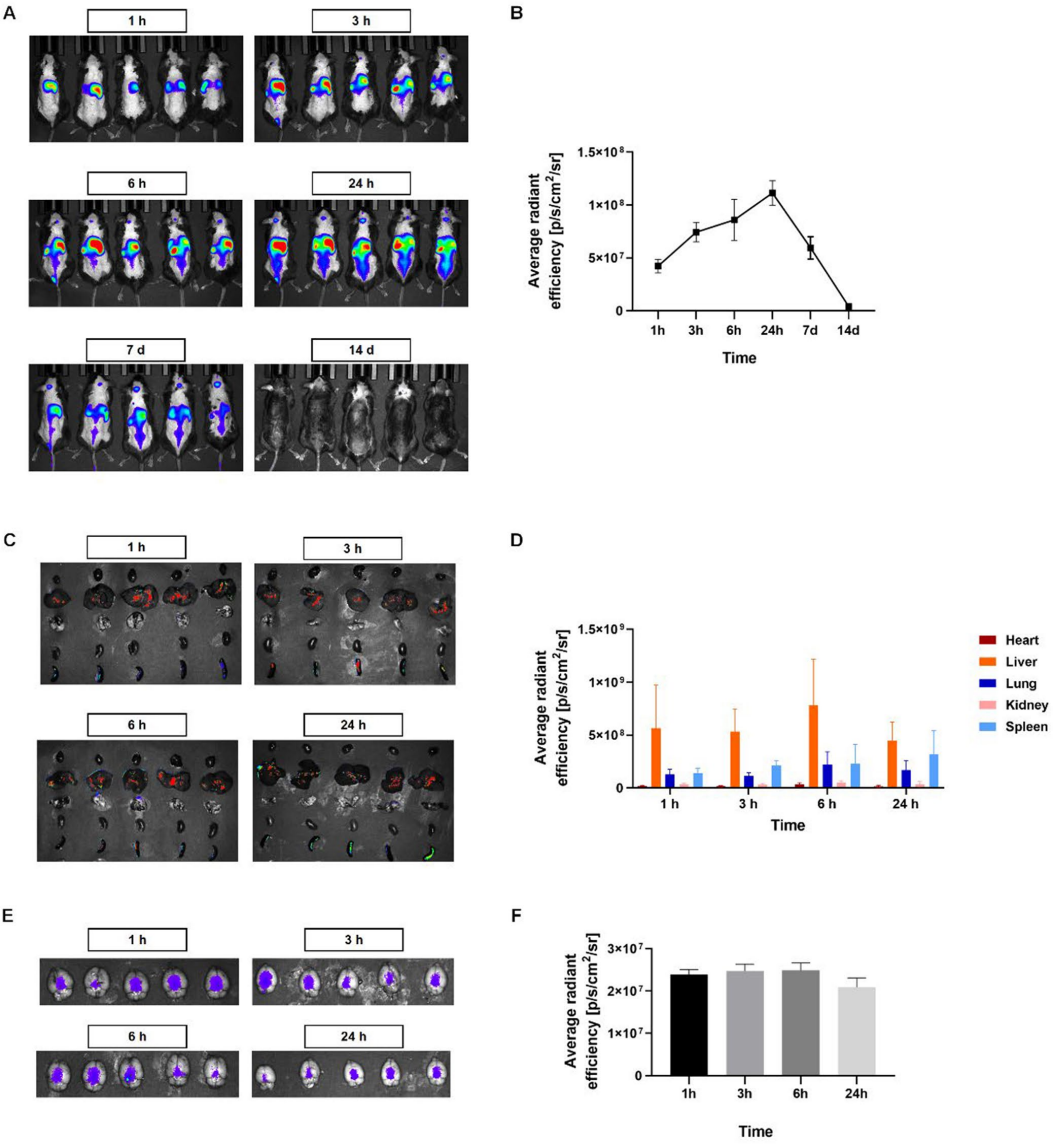
IMRC‐Exo biodistribution in mice imaged using the AniView multimodal imaging system. (A) IMRC‐Exo biodistribution in the whole body (*n* = 5). (B) Time‐course distribution of IMRC‐Exos in the whole body (*n* = 5). (C) IMRC‐Exo biodistribution in the heart, liver, lung, kidney, and spleen (*n* = 5). (D) Time‐course distribution of IMRC‐Exos in the heart, liver, lung, kidney, and spleen (*n* = 5). (E) IMRC‐Exo biodistribution in the brain (*n* = 5). (F) Time‐course distribution of IMRC‐Exos in the brain (*n* = 5). Data are presented as means ± SDs.

To further investigate the in vivo distribution of these nanovesicles, fluorescently labelled IMRC‐Exos were intravenously administered into mice via tail vein injection. Using the AniView multimodal imaging system, we noted that these nanovesicles were distributed in multiple organs within 1 h, peaked at 24 h, with a half‐life of 7 days. Notably, after 14 days, these exosomes were significantly metabolised and lost their biological efficacy. Moreover, the brain, heart, lung, liver, kidney, and spleen of mice were harvested and underwent ex vivo fluorescence imaging. Significant IMRC‐Exo distribution in all of these organs emerged, with more extensive distribution in the liver, spleen, and kidney, whereas the distribution levels remained relatively stable across the brain, heart, lung, liver, kidney, and spleen within 24 h (Figure 4). Fluorescence imaging on the brain tissue alone indicated that IMRC‐Exos could penetrate brain tissues, peaked at 3 h, and maintained distribution for at least 24 h.

### 3.3 Short‐Term Efficacy of IMRC‐Exo

**FIGURE 5:**
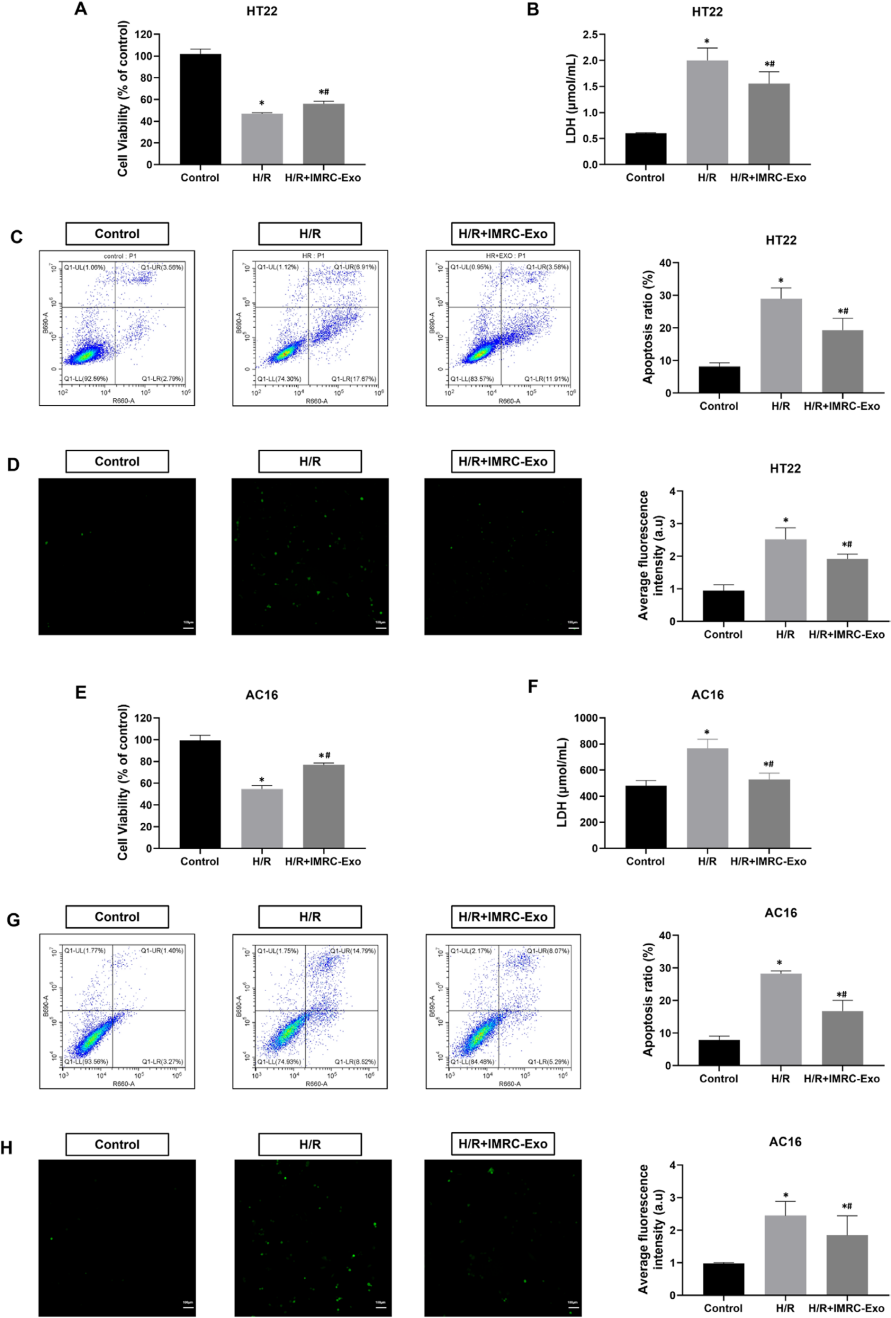
Effects of IMRC‐Exo on cell damage following H/R in HT22 and AC16 cells (A) Cell viability evaluated using a cell counting kit‐8 assay in HT22 cells (*n* = 3). (B) LDH levels detected using an LDH assay kit in HT22 cells (*n* = 6). (C) Flow cytometric analysis and quantification of cell apoptosis in HT22 cells (*n* = 3). (D) Representative fluorescence images and quantification of ROS production in HT22 cells (*n* = 3). (E) Cell viability evaluated using a cell counting kit‐8 assay in AC16 cells (*n* = 3). (F) LDH level detected using an LDH assay kit in AC16 cells (*n* = 6). (G) Flow cytometric analysis and quantification of cell apoptosis in AC16 cells (*n* = 3). (H) Representative fluorescence images and quantification of ROS production in AC16 cells (*n* = 3). Data are presented as means ± SDs. *p < 0.05 versus the control group; ^#^ *p* < 0.05 versus H/R.

To confirm the efficacy of IMRC‐Exos, systematic research was conducted across three levels as follows: cell, rat, and swine models. In the cell experiments, cells in the H/R and H/R + IMRC‐Exo groups demonstrated reduced activities compared with those in the control group (all *p* < 0.05). Notably, IMRC‐Exos significantly enhanced the viability of cells undergoing the H/R treatment (all *p* < 0.05). Additionally, compared with the control group, the H/R and H/R + IMRC‐Exo groups exerted significantly enhanced levels of LDH, ROS, and apoptosis ratio. Nevertheless, we also observed that cells receiving H/R + IMRC‐Exo treatment exhibited lower levels of LDH, ROS, and apoptosis ratio compared with those in the H/R group (Figure 5).

**FIGURE 6:**
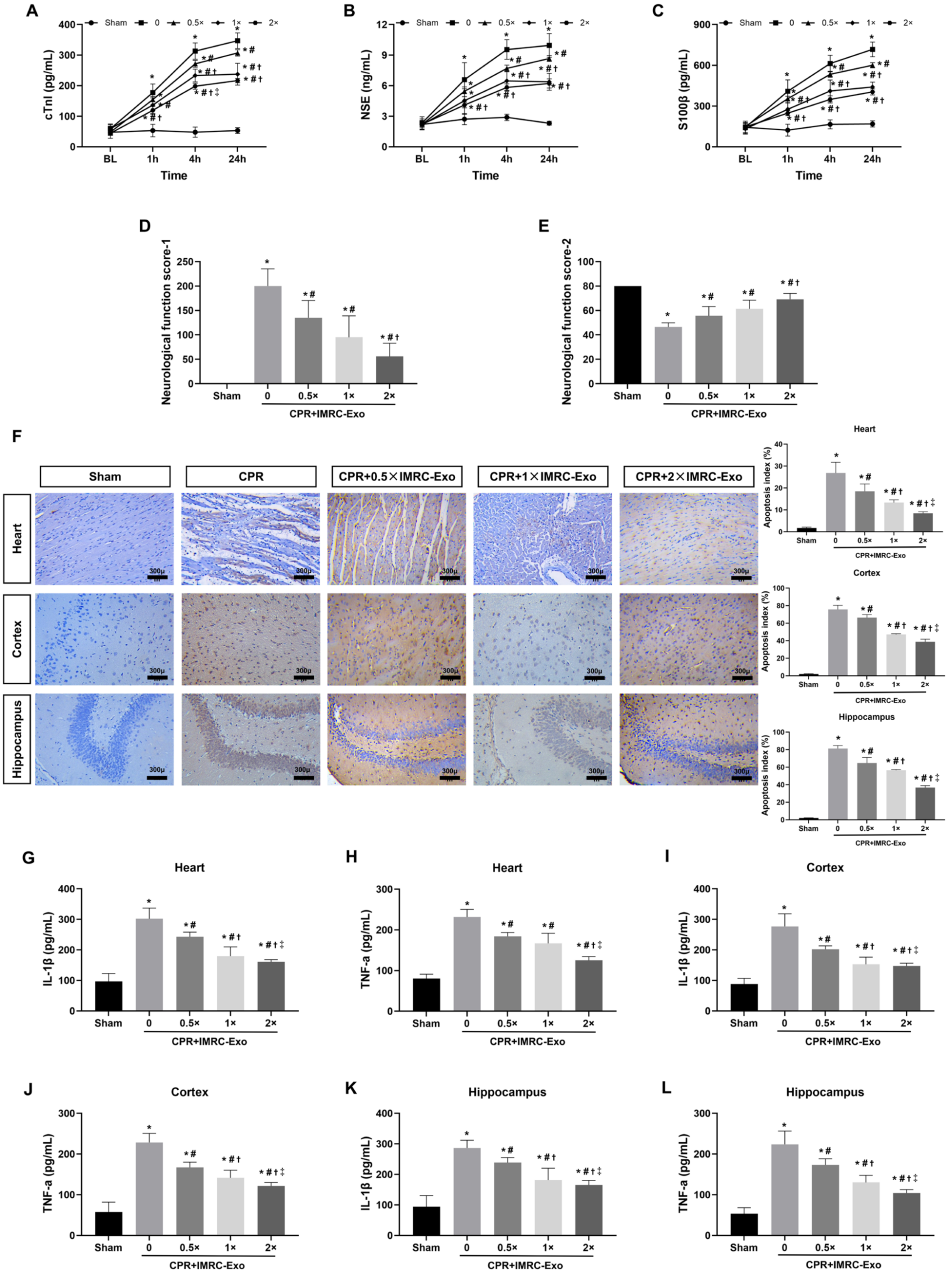
Effects of IMRC‐Exo on cardiac and cerebral injuries following CPR in rats. (A–C) cTnI, NSE, and S100β evaluated using ELISA at baseline and 1, 4, 6, and 24 h following resuscitation. (D, E) NFS‐1 and NFS‐2 evaluated at 24 h following resuscitation. (F) Representative photomicrographs of TUNEL assay (200×) and percentage of TUNEL‐positive cells in the heart, cortex, and hippocampus at 24 h following resuscitation. (G–L) IL‐1β and TNF‐α levels in the heart, cortex, and hippocampus at 24 h following resuscitation measured using ELISA (*n* = 5). 0.5×, 1.25 × 10^10^; 1×, 2.5 × 10^10^, 2×, 5.0 × 10^10^ particles/kg. Data are presented as means ± SDs. *p < 0.05 versus the sham group; ^#^ *p* < 0.05 versus the CPR group; ^†^ *p* < 0.05 versus the CPR + 0.5 × IMRC‐Exo group; ^‡^ *p* < 0.05 versus the CPR + 1 × IMRC‐Exo group.

Regarding rat models, 25 experiments were successfully conducted and completed. The results of baseline measurements, including hemodynamics, and the biomarkers of cardiac and cerebral injuries showed no significant difference across the five groups before CA (Figure S3A–F). During post‐resuscitation observation, all resuscitated animals exhibited significantly elevated cTnI, NSE, and S100β serum levels at various time points compared with the sham group. However, rats in the CPR + IMRC‐Exo group showed reduced cTnI, S100β, and NSE levels compared with the CPR group. Serum markers tended to decrease across the 0.5×, 1×, and 2× IMRC‐Exo groups. At 24 h post‐resuscitation, NFS‐1 scores were significantly increased and NFS‐2 scores were notably decreased in all resuscitated animals relative to the sham group. By contrast, IMRC‐Exo treatment significantly restored both NFS‐1 and NFS‐2 scores. In addition, IMRC‐Exo alleviated neurological dysfunction gradually with increasing doses. Furthermore, compared with the sham group, all resuscitated animals showed enhanced cell apoptosis within the cardiac and brain tissues. However, the CPR + IMRC‐Exo group presented a significantly lower apoptosis index than the CPR group. Crucially, the anti‐apoptotic effect was enhanced in a dose‐dependent manner. Similarly, compared with the sham group, all resuscitated rats demonstrated elevated TNF‐α and IL‐1β levels in cardiac and brain tissues, whereas these indicators were significantly reduced in the CPR + IMRC‐Exo group when compared with the CPR group. The changes presented a clear dose‐dependent relationship (Figure 6).

**FIGURE 7:**
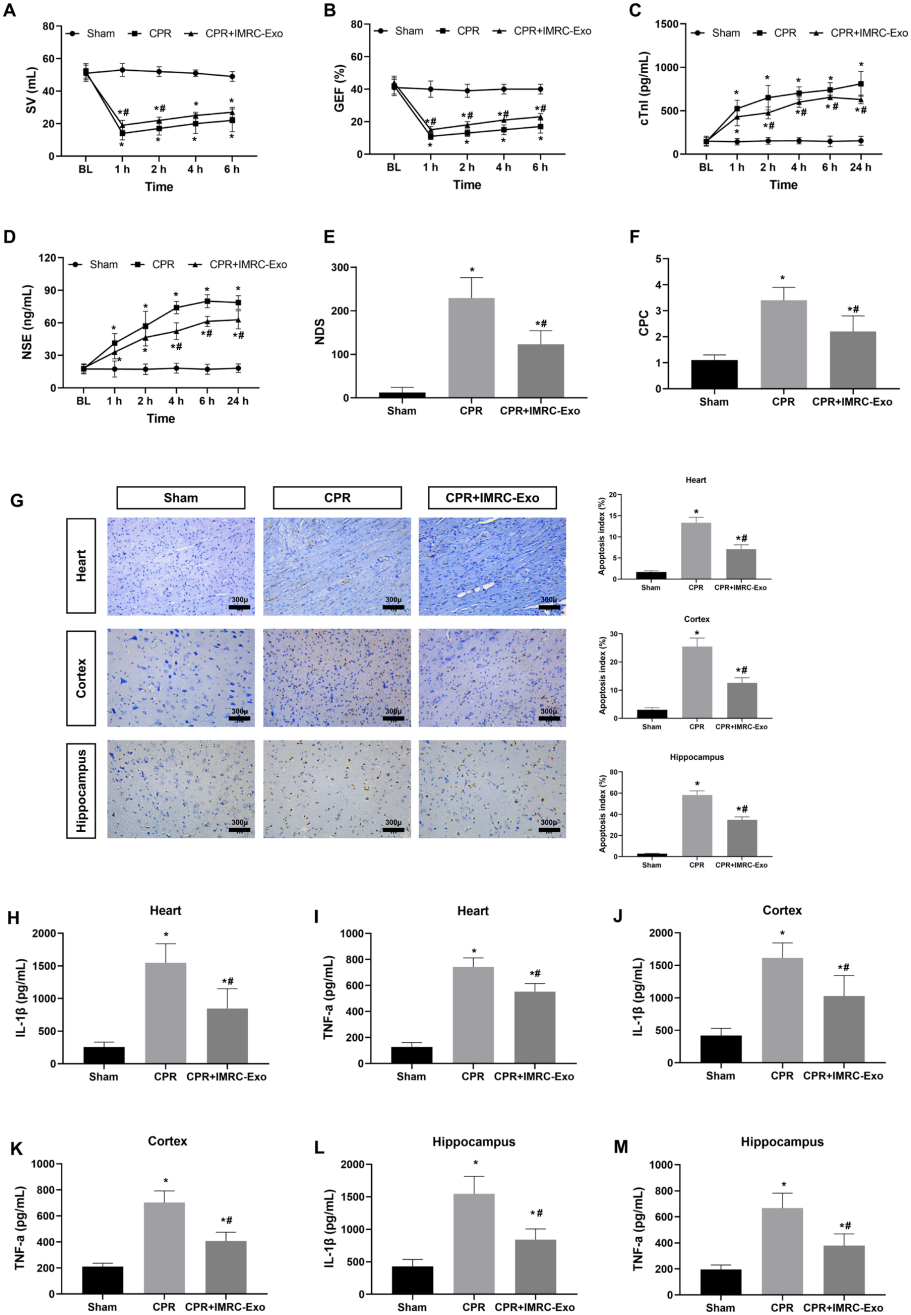
Effects of IMRC‐Exo on cardiac and cerebral injuries following CPR in swine. (A–D) SV and GEF evaluated using the PiCCO system, along with cTnI and NSE evaluated using ELISA at baseline and 1, 2, 4, 6, and 24 h following resuscitation. (E, F) NDS and CPC evaluated at 24 h following resuscitation. (G) Representative photomicrographs of TUNEL assay (200×) and percentage of TUNEL‐positive cells in the heart, cortex, and hippocampus at 24 h following resuscitation. (H–M) IL‐1β and TNF‐α levels in the heart, cortex, and hippocampus at 24 h following resuscitation measured using ELISA (*n* = 6). Data are presented as means ± SDs. *p < 0.05 versus the sham group; ^#^ *p* < 0.05 versus the CPR group.

Regarding swine models, 18 experiments were conducted and completed. The results of baseline examinations, including hemodynamics, blood gas, lactate, and cardiac and cerebral function, as well as injury biomarkers before CA, indicated no statistically significant differences (Figure S3G–O). During post‐resuscitation observation, all resuscitated animals showed significantly decreased SV and GEF levels, along with increased cTnI and NSE levels, compared with the sham group. However, swine in the CPR + IMRC‐Exo group exhibited significantly enhanced SV levels at 1 and 2 h post‐resuscitation and increased GEF values at all‐time points following resuscitation compared with the CPR group, whereas the cTnI and NSE levels substantially decreased at 4, 6, and 24 h following resuscitation. At 24 h post‐resuscitation, all resuscitated animals presented markedly elevated NFS exceeding the sham group, as assessed using NDS and CPC scores. However, IMRC‐Exo administration significantly weakened NDS and CPC scores. Furthermore, compared with the sham group, obvious cell apoptosis was noted within the cardiac and brain tissues of all resuscitated swine, whereas swine in the CPR + IMRC‐Exo group exhibited a significantly reduced apoptosis index. Consistent with apoptosis, the levels of the pro‐inflammatory factors (TNF‐α and IL‐1β) within the cardiac and brain tissues of resuscitated swine were significantly increased. Notably, CPR + IMRC‐Exo treatment markedly reduced TNF‐α and IL‐1β levels (Figure 7).

### 3.4 Long‐Term Protective Efficacy of IMRC‐Exo

As expected, 100% of rats in the sham group survived until the end of the study. In contrast, the CPR group showed a marked decline in survival, with a final 7‐day survival rate of 50%, and all mortalities occurred within the first 4 days after resuscitation. The CPR + IMRC‐Exo group demonstrated an improved 7‐day survival rate of 83.3% compared with the CPR group. However, the log‐rank test revealed no statistically significant difference in survival between the CPR and CPR + IMRC‐Exo groups.

At 7d post‐resuscitation, we observed that the NFS‐1 scores were significantly elevated, and the NFS‐2 scores were markedly depressed in all resuscitated animals compared with those in the sham group, whereas rats receiving IMRC‐Exo treatment exhibited drastically enhanced NFS‐1 and NFS‐2 scores (Figure S4).

### 3.5 Protective Mechanism of IMRC‐Exo

**FIGURE 8:**
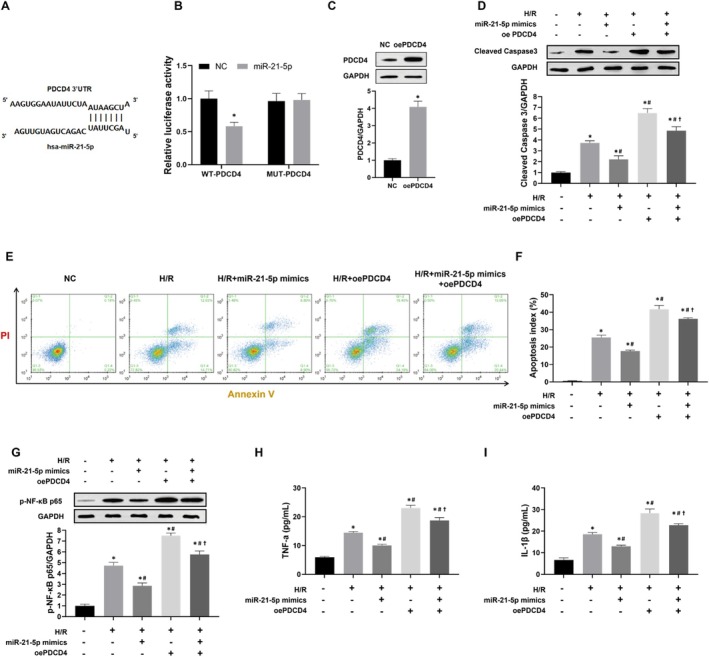
miR‐21‐5p mimics alleviate H/R‐induced apoptosis and inflammatory response in AC16 cells by targeting and inhibiting PDCD4. (A) Diagram showing the binding site of miR‐21‐5p in the 3′UTR region of the PDCD4 gene. (B) Luciferase reporter assay and analysis of the binding between miR‐21‐5p and PDCD4. (C) Relative expression levels of PDCD4 in AC16 cells transfected with PDCD4 overexpression plasmid. (D) Expression levels of Cleaved Caspase 3 in AC16 cells at 24 h after H/R stimulation. (E, F) Flow cytometric analysis and quantification of apoptosis in AC16 cells at 24 h after H/R stimulation. (G) Expression levels of p‐NF‐κB p65 in AC16 cells at 24 h after H/R stimulation. (H) Levels of TNF‐α in the supernatant of AC16 cells at 24 h after H/R stimulation. (I) Levels of IL‐1β in the supernatant of AC16 cells at 24 h after H/R stimulation. Each group included three replicates. Data are presented as means ± SDs. *p < 0.05 versus NC; ^#^ *p* < 0.05 versus H/R; ^†^ *p* < 0.05 versus H/R + miR‐21‐5p mimics.

**FIGURE 9:**
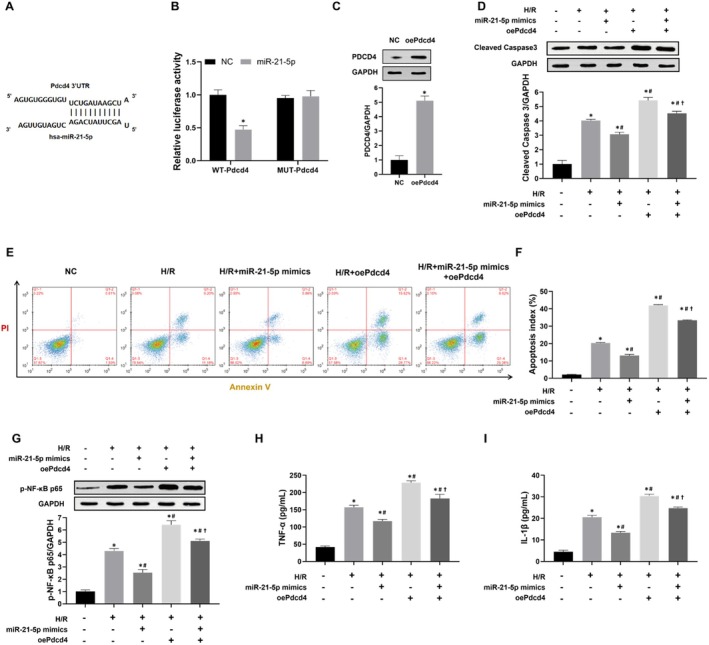
miR‐21‐5p mimics alleviate H/R‐induced apoptosis and inflammatory response in HT22 cells by targeting and inhibiting PDCD4. (A) Diagram showing the binding site of miR‐21‐5p in the 3′UTR region of the PDCD4 gene. (B) Luciferase reporter assay and analysis of the binding between miR‐21‐5p and PDCD4. (C) Relative expression levels of PDCD4 in HT22 cells transfected with PDCD4 overexpression plasmid. (D) Expression levels of Cleaved Caspase 3 in HT22 cells at 24 h after H/R stimulation. (E, F) Flow cytometric analysis and quantification of apoptosis in HT22 cells at 24 h after H/R stimulation. (G) Expression levels of p‐NF‐κB p65 in HT22 cells at 24 h after H/R stimulation. (H) Levels of TNF‐α in the supernatant of HT22 cells at 24 h after H/R stimulation. (I) Levels of IL‐1β in the supernatant of HT22 cells at 24 h after H/R stimulation. Each group included three replicates. Data are presented as means ± SDs. Data are presented as means ± SDs. *p < 0.05 versus NC; ^#^ *p* < 0.05 versus H/R, ^†^ *p* < 0.05 versus H/R + miR‐21‐5p mimics.

In our previous study, we performed sequencing analysis of miRNAs in IMRC‐Exo, and the results showed that miR‐21‐5p was the most abundant [18]. To further clarify its protective mechanism, TargetScan prediction indicated that miR‐21‐5p could target the 3′UTR of human PDCD4 and mouse Pdcd4, with the seed sequence binding sites shown in Figures 8A and 9A. Dual‐luciferase reporter assay confirmed that co‐transfection with miR‐21‐5p mimics significantly reduced the luciferase activity of wild‐type PDCD4/Pdcd4 reporters, whereas no significant change was observed in the mutant type, suggesting that miR‐21‐5p directly targets and inhibits PDCD4/Pdcd4 expression (Figures 8B and 9B).

Subsequently, PDCD4 overexpression models were established in AC16 and HT22 cells, respectively (Figures 8C and 9C), followed by treatment with miR‐21‐5p mimics. Compared with the NC group, the protein levels of Cleaved Caspase‐3, apoptotic index, protein level of p‐NF‐κB p65, and levels of TNF‐α and IL‐1β were significantly increased in the H/R and each intervention group (Figures 8D–I and 9D–I). Compared with the H/R group, these indicators were markedly decreased in the H/R + miR‐21‐5p mimics group, indicating that miR‐21‐5p mimics significantly alleviated H/R‐induced apoptosis and inflammation. In contrast, Cleaved Caspase‐3 expression, apoptotic index, p‐NF‐κB expression, and inflammatory cytokine levels were further significantly elevated in the H/R + oePDCD4 group, suggesting that PDCD4 overexpression aggravated H/R‐induced cell injury. Meanwhile, the protective effect of miR‐21‐5p mimics was significantly abolished by PDCD4 overexpression in the H/R + miR‐21‐5p mimics + oePDCD4 group compared with the H/R + miR‐21‐5p mimics group.

## 4 Discussion

This study represented a systematic preclinical research assessing the safety, pharmacokinetic characteristics, and efficacy of IMRC‐Exo in the model of CA and resuscitation, filling the gap in this field. These aforementioned results indicated that in both in vitro and in vivo experiments, IMRC‐Exos did not induce distinct apoptosis or oxidative damage and maintained routine blood indices within the normal ranges, with no histopathological alterations observed. Furthermore, pharmacokinetic studies demonstrated that IMRC‐Exos could rapidly reach major organs throughout the body, reaching peak concentrations in brain tissues at 3 h post‐administration. Notably, in rat models, the protective effects of IMRC‐Exo exhibited a dose‐dependent manner, with therapeutic benefits markedly enhanced as the intervention concentration increased. Moreover, long‐term observational results confirmed that IMRC‐Exo significantly improved neurological function and survival rate at 7 days after treatment. Mechanistically, miR‐21‐5p derived from IMRC‐Exo directly targets PDCD4, thereby alleviating cellular apoptosis and inflammatory responses. Therefore, these comprehensive data paved the way for advanced clinical studies on exosomes as promising therapeutic agents.

MSC‐Exo, as an emerging therapeutic formulation, has demonstrated remarkable potential in the management of diseases, including hemorrhagic shock (HS) and I/R injury. Several studies have indicated that MSC‐Exos can alleviate myocardial I/R injury in mice by modulating neutrophil infiltration and neutrophil extracellular trap formation [24]. Moreover, MSC‐Exos can ameliorate cerebral I/R injury by regulating microglial polarisation to inhibit inflammation and pyroptosis mediated by NLR family pyrin domain containing 3 (NLRP3) inflammasomes [25]. In large animal models of traumatic brain injury (TBI) and HS, early single‐dose treatment with MSC‐Exos significantly reduced brain swelling and lesion sizes, decreased cerebral biomarker levels, inhibited inflammation and apoptosis, improved blood–brain barrier integrity, promoted neural plasticity, and ameliorated long‐term neurological outcomes [26, 27, 28]. Although the protective effects of IMRC‐Exos on the heart and brain in CA models have not been substantiated, increasing evidence has revealed that exosomes carrying various miRNA can protect against cardiac and cerebral injuries. For instance, miR‐21‐5p, miR‐22‐3p, miR‐31, and miR‐145 have been shown to target Fas Ligand, lysine demethylase 6B, tumour necrosis factor receptor‐associated factor 6, interferon regulatory factor 5, and Forkhead box O1, resulting in the inhibition of neuronal apoptosis and improvement of neurological function [29, 30, 31, 32]. Furthermore, miR‐29c, miR‐144, and miR‐486‐5 can target phosphatase and tensin homologue to inhibit cardiomyocyte apoptosis under hypoxic conditions [33, 34, 35]. Based on this finding, it is indispensable to further delve into the therapeutic efficacy of these nanovesicles in CA models.

To date, published clinical studies to date have reported good safety profiles [36, 37, 38, 39], endowing a solid scientific basis for our safety assessment. Previous studies have reported that the administration of 3 × 10^9^–5 × 10^10^ exosome particles via tail vein into mice did not cause any significant toxicity [36, 37]. Based on these findings, healthy mice underwent intravenous injection via tail vein with 5 × 10^8^, 1 × 10^9^, 2.5 × 10^9^, and 5 × 10^9^ particles to assess IMRC‐Exo toxicity. The biosafety results indicated that IMRC‐Exo injection had no prominent effect on body weight or hematologic parameters. Moreover, IMRC‐Exo did not induce inflammatory cell infiltration and substantial tissue damage in any main organs. These findings further confirmed the in vivo biological safety of IMRC‐Exo, aligning with previous assessments of exosomal safety, despite the varying sources of exosomes [38, 39, 40, 41]. In conclusion, these consistent results highlighted the safety of IMRC‐Exo as a promising therapeutic agent.

Pharmacokinetics, a major factor in assessing the safety and efficacy of drugs, has been regarded as a pivotal indicator for clinical drug utilisation. In this context, we selected a concentration of 5 × 10^8^ particles to observe the distribution and metabolic patterns of IMRC‐Exo in mice based on previous literature. Notably, we noted that IMRC‐Exos were distributed in various organs, including brain tissues, within 1 h, peaking at 6 h in the heart, lung, liver, and kidney, and at 3 h in the brain and spleen. These findings align with previous studies [40, 42]; however, previous studies have indicated that these nanovesicles reached their peak distribution within the brain, kidney, liver, and lung at 2 h post‐injection. One possible reason for this differentiated phenomenon may be the distinct fluorescent labeling methods employed. Specifically, in this study, we labelled IMRC‐Exos with exosome fluorescent labeling dye, whereas Hojun Choi et al. labelled exosomes with zirconium‐89. Moreover, we utilised fluorescence imaging technology in our experiments, whereas Hojun Choi et al. employed positron emission tomography for imaging [42]. These technical differences may have impacted the observation and interpretation of the biodistribution characteristics of exosomes. Additionally, the source of exosomes can affect their distribution in the body [43]. Overall, this study revealed the distribution characteristics of IMRC‐Exos in mice, confirming that IMRC‐Exo can penetrate multiple major organs, including the heart, lung, liver, kidney, and brain, providing crucial foundational data for clinical applications.

Efficacy was a core indicator for evaluating the clinical application value of drugs. Existing research has demonstrated that exosomes possessed significant biological activity within a specific concentration range and exhibited therapeutic effects in various diseases. In preclinical studies, particularly in large animal models of TBI and HS, the dosage range of 1 × 10^10^–1 × 10^13^ particles of exosomes can inhibit inflammation and cell apoptosis, significantly ameliorating the severity of neurological damage [27, 28]. Therefore, an intermediate dose of 2.5 × 10^10^ particles/kg of IMRC‐Exos was selected in the present study. Compared with the control group, IMRC‐Exo–treated rats demonstrated significantly improved cardiac and cerebral dysfunction and injuries, accompanied by substantially decreased cardiac and cerebral apoptosis. This finding aligned with previous studies, supporting the potential of exosomes as a new‐generation therapy for improving cardiac and cerebral injuries following CA and providing a firm scientific basis for future clinical applications.

In the present study, we further explored the potential molecular mechanism by which IMRC‐Exo alleviates H/R‐induced injury in vitro. Previous studies showed MSC‐derived exosomes exert biological functions mainly by delivering abundant functional miRNAs to recipient cells [44]. Our previous sequencing results confirmed that miR‐21‐5p is one of the most enriched miRNAs in IMRC‐Exo [18]. Accumulating evidence has demonstrated that miR‐21‐5p participates in the regulation of apoptosis and inflammatory responses by targeting downstream genes [45, 46]. Meanwhile, PDCD4 acts as a pro‐apoptotic and pro‐inflammatory molecule. Upregulated PDCD4 expression exacerbates ischemia/reperfusion injury, and PDCD4 possesses potential binding sequences for miR‐21‐5p [47, 48, 49]. In this study, we validated that miR‐21‐5p directly targets and inhibits PDCD4 expression. Furthermore, rescue experiments demonstrated the miR‐21‐5p mimics obviously reduced excessive apoptosis and inflammatory response in H/R‐injured HT22 and AC16 cells, which simulated the protective effect of IMRC‐Exo intervention. In contrast, PDCD4 upregulation partially reversed the beneficial effects of miR‐21‐5p. These data demonstrated that miR‐21‐5p attenuates H/R‐induced apoptosis and inflammation by targeting and inhibiting PDCD4 in both AC16 and HT22 cells.

Although promising, this study also had several limitations. First, our study demonstrated improved 7‐day survival and neurological function in rats compared with the CPR group, but whether these effects can be reproduced in swine remains to be determined. Second, the biodistribution of IMRC‐Exo was only studied in mice, while its biodistribution in swine remains to be further investigated. Third, the molecular mechanism was only verified in vitro. Further in vivo intervention experiments focusing on the miR‐21‐5p/PDCD4 pathway will be conducted in subsequent research to enrich the mechanistic evidence of IMRC‐Exo for PCAS treatment.

## 5 Conclusion

This study revealed that in the preclinical treatment of PCAS, IMRC‐Exo was a safe and effective therapeutic agent, rapidly distributing from the bloodstream to target organs, including the heart and brain, providing protective benefits for post‐resuscitation cardiac and cerebral injuries.

## Author Contributions

**Wenbin Zhang:** conceptualization, writing – original draft, methodology, validation, data curation. **Ziwei Chen:** data curation, resources. **Jinyu Zhu:** data curation, resources. **Yufeng Hu:** data curation, resources. **Mao Zhang:** conceptualization, methodology, writing – review and editing, project administration, funding acquisition. **Huijuan Yang:** methodology, validation, formal analysis, data curation, investigation, writing – original draft. **Licai Liang:** data curation, resources. **Xue Wang:** methodology, validation, writing – original draft, investigation. **Lu He:** data curation, resources. **Jie Wang:** data curation, resources. **Jiefeng Xu:** conceptualization, methodology, writing – review and editing, project administration, funding acquisition. **Xiaodan Zhang:** investigation, writing – original draft, methodology, validation, data curation.

## Funding

This work was supported by the Zhejiang Provincial Key Research and Development Program of China (2024C04045, 2021C03073), the Natural Science Foundation of China (82372204, 82472234).

## Disclosure

*Declaration of generative AI and AI‐assisted technologies in the writing process*: During the preparation of this work, the authors utilised AI to assist with grammar refinement. The authors subsequently reviewed and edited the content as needed and take full responsibility for the final version of the manuscript.

## Ethics Statement

This animal study was approved by the Institutional Ethics Committee of the Second Affiliated Hospital of Zhejiang University School of Medicine. Project title 1 (for swine study): Study on the Efficacy and Mechanisms of MSC‐Derived Exosomes in Alleviating Post‐Resuscitation Multiple Organ Injury in a Swine Model of Cardiac Arrest (approval number, 2023‐064; date of approval, May 19, 2023). Project title 2 (for rat study): Role and Mechanism of hESC‐Derived MSCs in Protecting Cardiac and Cerebral Injury in Rats with Cardiac Arrest (approval number, 2024‐169; date of approval, September 13, 2024). Project title 3 (for mice study): Study on the Safety, Pharmacokinetics, and Efficacy of hESC‐IMRC‐Exo in the Treatment of Cardiac and Cerebral Injury Post‐Cardiac Arrest Resuscitation (approval number, 2025‐104; date of approval, April 18, 2025). Methods for each procedure were performed in accordance with the approved guidelines and regulations. The manuscript adheres to the ARRIVE guidelines (https://arriveguidelines.org/arrive‐guidelines) for the reporting of animal experiments.

## Consent

The authors have nothing to report.

## Conflicts of Interest

The authors declare no conflicts of interest.

## Supporting information


**Figure S1:** Schematic representation of 3′‐UTR of human PDCD4 mRNA reporter with and without the miR‐21‐5p seed‐binding site (red).

**Figure S2:** Schematic representation of 3′‐UTR of mouse Pdcd4 mRNA reporter with and without the miR‐21‐5p seed‐binding site (red).

**Figure S3:** Baseline and CA/CPR characteristics in Rats and Swine studies.

**Figure S4:** Long‐term protective efficacy of IMRC‐Exo.

## Data Availability

The manuscript and Supporting Information contain all the data collected during the current study, and the corresponding author can provide unprocessed data upon reasonable request.
