# Supplementary material for: Assessment of hESC‐IMRC‐Exo for Cardiac and Cerebral Injuries Post‐Cardiac Arrest Resuscitation: Safety, Pharmacokinetics, and Efficacy
Source: J Cell Mol Med. 2026 Jun 26;30(12):e71264. doi: 10.1111/jcmm.71264 (PMC13309394; doi:10.1111/jcmm.71264)
Supplement: Supplementary file 1 — Figure S1: Schematic representation of 3′‐UTR of human PDCD4 mRNA reporter with and without the miR‐21‐5p seed‐binding site (red). [file JCMM-30-e71264-s003.docx]

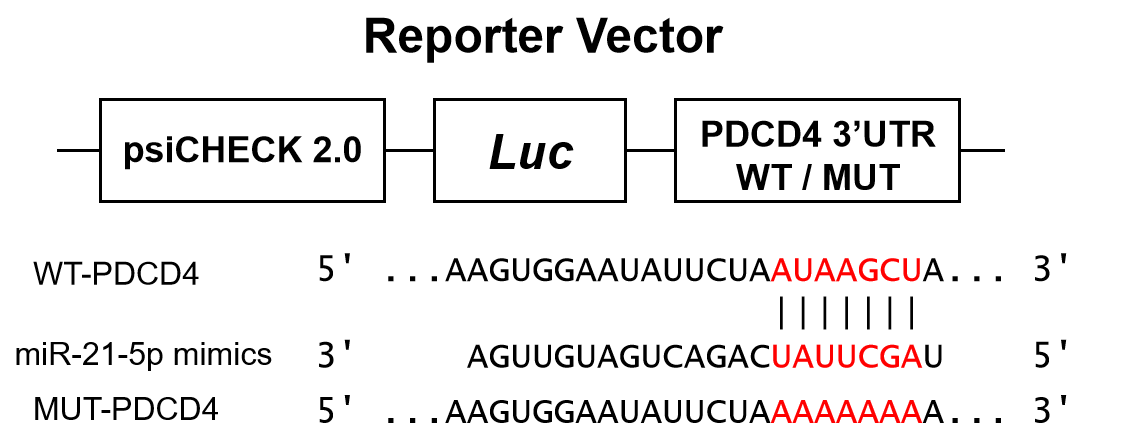


**Figure S1. Schematic representation of 3′-UTR of human PDCD4 mRNA reporter with and without the miR-21-5p seed-binding site (red)**
