# Supplementary material for: Assessment of hESC‐IMRC‐Exo for Cardiac and Cerebral Injuries Post‐Cardiac Arrest Resuscitation: Safety, Pharmacokinetics, and Efficacy
Source: J Cell Mol Med. 2026 Jun 26;30(12):e71264. doi: 10.1111/jcmm.71264 (PMC13309394; doi:10.1111/jcmm.71264)
Supplement: Supplementary file 3 — Figure S3: Baseline and CA/CPR characteristics in Rats and Swine studies. [file JCMM-30-e71264-s004.docx]

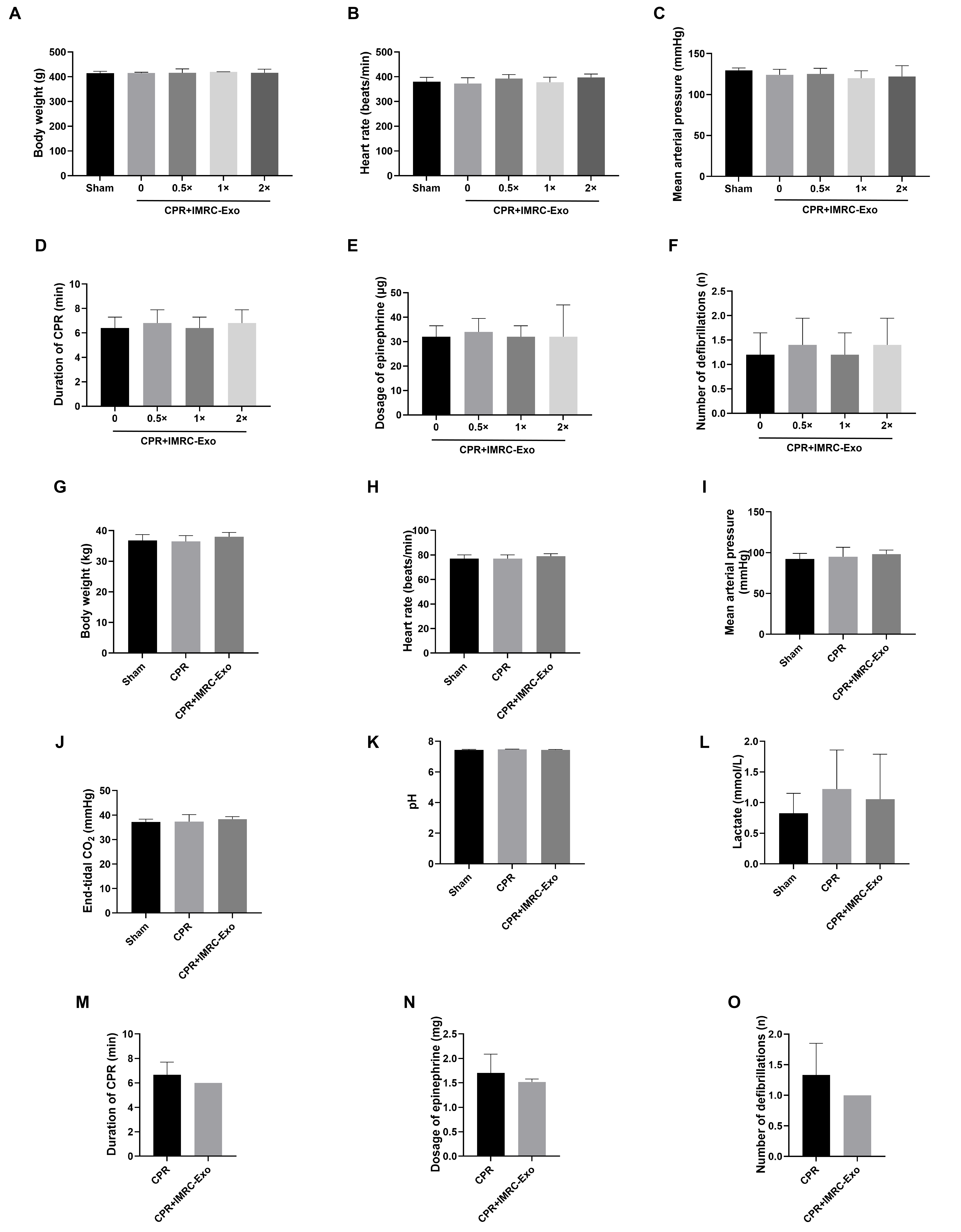


**Figure S3. Baseline and CA/CPR characteristics in Rats and Swine studies.**

1. **F** Baseline and CA/CPR characteristics including body weight, heart rate, mean arterial pressure, the duration of CPR, the number of defibrillations performed, and the cumulative dose of epinephrine administered assigned to the sham group, CPR, and CPR+IMRC-Exo group in rats(n=5). **G-O** Baseline and CA/CPR characteristics including body weight, heart rate, mean arterial pressure, end-tidal carbon dioxide, pH, lactate, the duration of CPR, the number of defibrillations performed, and the cumulative dose of epinephrine administered assigned to the sham group, CPR, and CPR+IMRC-Exo group in swine(n=6). 0.5×, 1.25×10^10^; 1×, 2.5×10^10^, 2×, 5.0×10^10^ particles/kg. Data are presented as means ± SDs. *p < 0.05 versus the sham group; #p < 0.05 versus the CPR group; †p < 0.05 versus the CPR+0.5×IMRC-Exo group; ‡p < 0.05 versus the CPR+1×IMRC-Exo group.
