# Supplementary material for: Assessment of hESC‐IMRC‐Exo for Cardiac and Cerebral Injuries Post‐Cardiac Arrest Resuscitation: Safety, Pharmacokinetics, and Efficacy
Source: J Cell Mol Med. 2026 Jun 26;30(12):e71264. doi: 10.1111/jcmm.71264 (PMC13309394; doi:10.1111/jcmm.71264)
Supplement: Supplementary file 4 — Figure S4: Long‐term protective efficacy of IMRC‐Exo. [file JCMM-30-e71264-s002.docx]

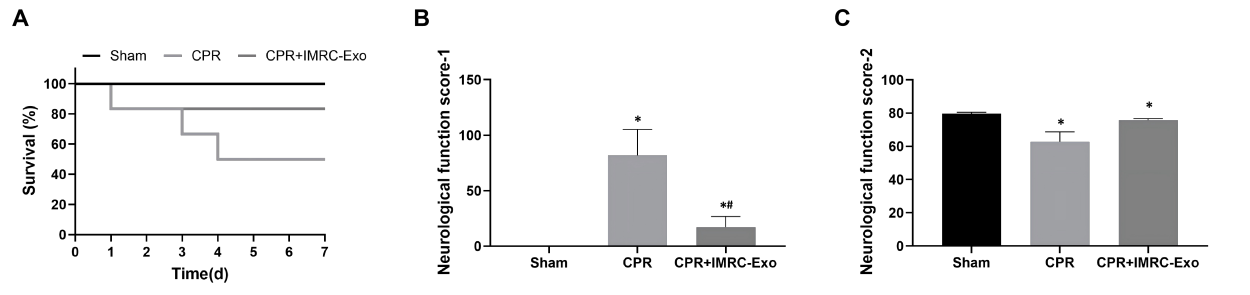


**Figure S4. Long-term protective efficacy of IMRC-Exo**

Data are presented as means ± SDs. *p < 0.05 versus the sham group; #p < 0.05 versus the CPR group.
